# Dentists and the Census of 1890. Historical Sketch

**Published:** 1895-03

**Authors:** Richard Grady


THE
### AMERICAN JOURNAL
—OF—
### DENTAL SCIENCE.

VOL. XXVIII. BALTIMORE, MARCH, 1895. NO. 11.

ARTICLE I.

### DENTISTS AND THE CENSUS OF 1890. HISTORICAL SKETCH.

BY RICHARD GRADY, M. D., D. D. S.

*Dr. Alonzo Boice, Chairman, etc., American Dental Association:*

You did me the honor to say recently to the Governor of Maryland that I was the first dentist in the country to protest in 1890 against the classification of members of the dental profession as manufacturers. It may be that I was called into wakefulness while others were slumbering.

As the question of priority has enlisted some zeal without definite settlement I shall, in complying with your request to tell what I know of the opposition, confine myself with all consideration and courtesy to the time previous to the passage, June 4, 1892, of the Willcox Bill which provided penalties for refusal to answer questions.

Everything has a beginning and an end; and, when I heard that success had crowned the ability and the energy of the Associated Committees of the Dental Societies, June

27, 1892, nearly two years (fully 22 months) after I had first protested, the words of Solomon recurred to my memory, "Better the end of a thing than the beginning." Whilst the inception, in the teeth of clinched antagonism, marks an era in the history of dentistry, for the final result I accord merited credit to the "associated committees" composed as they were of men resolutely and indignantly determined to use every means in their power to make Congress see the injustice of deposing dentists from their honorably acquired position of professional men.

As I take it, *the date of the first recorded protest* is the central question in any statement of the case. What I published in the *Baltimore News* under the caption, "Are Dentists Manufacturers?" is quoted at length as printed with my name and the date, September, 1, 1890. It is an historical fact beyond question which must never be lost sight of by your committee appointed to "draft a sketch of the history of dentists being classified as manufacturers by the Census Bureau of 1890."

### ARE DENTISTS MANUFACTURERS?—THE NEW CENSUS CREATING MORE TROUBLE AND VEXATION.

*Editor of the News*:

Dear Sir—An agent of the Census Department handed me last week a blank to be filled on the ground that dental offices are "establishments of productive industry," and that dentists are required to furnish statistics of manufactures to the census bureau. I represented to him that the dental surgeon cannot properly be subjected to investigation and annoyance in respect to his professional work any more than the surgeon in other specialties of the medical art, that he stands in delicate and confidential relations to his patients, that the demand for reports from dental practitioners being novel and unjustifiable, there would be general reluctance on the part of the profession to give the information contemplated by the schedule, and that the superintendent of the census of 1880, after correspondence, determined to omit from the tabulations of the office, "dentistry in all its branches," and had all the schedules on file endorsed to that effect and returned to their respective makers. With this explanation the agent left and next day called and took the blank away. Subsequently he returned with it and said that he was required to obtain the information necessary to fill the blank, which the law compelled me to give.

I am desirous to comply with every legal demand and also to save all connected with the census office from trouble. The schedule demonstrates, however, that the difficulty in the way is that the specialty of dental surgery is not comprehended, otherwise it would be seen that it is unwise, if not illegal, to demand statements from dental practitioners respecting their professional doings. The word surgery denotes hand-work, in connection with the art of healing, and this whether manual or instrumental, or both conjointly. Orthopedy (medical) is the art of curing or remedying deformities in the human body. Prothesis (surgical) signifies the addition of some artificial part to the human body. Marked examples of both these classes are shown in the report of the International Exhibition (1876) edited by Gen. Francis A. Walker, chief of the census office in 1880, for example:

"Then followed twenty-two specimens of *prothesis* and *orthopedy*, embracing pivot teeth, dentures, complete and incomplete, with and without spiral springs, the various methods of retention by atmospheric pressure and by clasps, and a series of orthopedic apparatus for the correction of congenital malformations of the palate, and loss by scrofulous and syphilitic necrosis."

Of the judges of the group on medicine, surgery and prothesis no one was a dentist; all were in medicine and surgery, and their testimony shows that the exhibits, which embraced or represented in principle all which dentists make or use in practice, were surgical.

The appliances made in the operating-rooms and laboratories of dentists, which the census office claim as "mechanical," are made only after examination of the patient, or of the mouth, or parts for which the appliance is needed. In some cases teeth or other parts require removal or some additions, such as fillings, alteration in form, extraction, the removal of diseased bone, etc., and the healing of the parts. In most cases the appliance can not be made except by the aid of casts or impressions, taken from each particular patient or person for whom the appliance is necessary, without whose co-operation the work can not begin. When the appliance is finished it is useless and of no value except to or for that *particular patient.* These appliances are thus surgical. They are the results of diagnosis, prognosis and hand-work combined to produce a useful result in, or appliance to, or for, the human frame; this even when no medicine is given or prescribed in surgery. In filling teeth the result of the dentist's labor is the addition of artificial parts to defective organs of the human body: this addition to each tooth of a patient being when completed virtually a part of that *particular person.* Filling teeth is therefore a surgical operation.

No schedule was left with physicians and surgeons; lawyers and other professional men were not asked to report; why, then, should dental practitioners be called upon to give information in regard to the personal treatment of their patients? Dentistry in *all* its branches was omitted from the tabulations of the census office in 1880, what is the reason for including it in 1890?

RICHARD GRADY, M. D., D. D. S.

Baltimore, Sept. 1, 1890.

The Association of Dental Surgeons of Baltimore City (incorporated) was the first dental organization to take formal action. A special meeting was called for September 12, 1890, "to determine whether as a body the Association should resist the demand of the census officers for an account of the profits of dentists from mechanical dentistry." (An original copy of the call for this meeting which I printed, dated September 11, 1890, is filed herewith, marked A.)

BALTIMORE, September 11, 1890.

*Dear Doctor:*

You are urged to be present at Dr. Volck's, 338 N. Charles street on Friday, Sept. 12th, at 5 p. m., to determine whether as a body the Association should resist the demand of the census officers for an account of the profits of dentists from "mechanical" dentistry.

By authority of the President, Dr. Volck,

W. S. TWILLEY, Secretary,
Association of Dental Surgeons.

The favorable action of the Association led the Chief

Special Agent at Baltimore, within a week, to write to the Census Office, Washington. He received this answer:

"Replying to your inquiry of September 18, 1890, referring to mechanical dentists, you are informed that the term is considered by this office to embrace all branches of the profession, except the extraction of teeth and should be duly reported on General Schedule, No. 3." (Copy herewith markedB.)

Having done what I could by appeals through the press, vigorous personal efforts, repeated again and again, and the influence of the Association of Dental Surgeons which I had founded in 1888, and realizing that the matter demanded the combined support of the dentists in the community, I enlisted the services of Dr. Wm. Shaw Twilley, active, zealous, acute, to whom I gave a copy of the letter last quoted, believing that he would command co-operation and helpfulness where I could not. He returned the letter October 1, 1890, and wrote: "Enclosed please find the letter, I showed same to Dr.——, and he promised to bring the matter up before 'the society', of which he is a member, on Thursday evening, the result of which I think will be a call for the profession to take some action in the matter. As soon as I had explained to him the questions asked he expressed his willingness to join in the fight and assist (*me* not you) all he could." (Original letter herewith marked C). Such, unhappily, is the truth of history. I do what seems to me right with the letter (omitting the name however) and with another dated November 22, 1890, to be quoted later.

October 3, 1890, the Chief Special Agent and the agent who left the blank in August, 1890, called at my office, but I was out of city.

October 6, 1890, I wrote the Census Special Agent regretting that I was not in when he called and that a committee had been appointed to confer with him and if necessary go to Washington, to which (October 7, 1890) he replied: "I would be pleased to confer with your committee at such time as will suit their convenience, provided it is at an early date as I cannot allow the work to be delayed." (Original letter herewith marked D.)

October 14, 1890, I wrote Dr. Thos. Brian Gunning, the following (original):

BALTIMORE, October 14, 1890.

*Dr. Thos. Brian Gunning:*

My Dear Sir:—The census department has given me until Friday to fill a blank on the ground that dental offices are "establishments of productive industry." The penalty for non-compliance, so the agent informs me, is a fine of $100.

Knowing that, owing to your prompt and earnest protest, the profession in New York, if not elsewhere, was spared in the last census, I write to ask if any demand has been made on the dental profession in your city for professional information for the Census of 1890 and your advice as to how I had best proceed in refusing to comply without incurring the legal penalty.

If you will mail me a few lines by to-morrow night, I will receive letter Thursday morning and have one day to determine my action.

With great respect,

RICHARD GRADY.

Miss Gunning has just received the enclosed through her lawyers. Dr. Thos. Brian Gunning died Jan. 8th, 1889.

It seems strange that the dental profession at large is not yet aware of his decease, and we are much annoyed by dental publications and periodicals regularly sent, though no payments have been made for them in five years, as Dr. Gunning on account of ill health retired from practice in March 1886, giving up his New York home the following July. Will you kindly do all in your power to make known Dr. Gunning's death?

October 22, 1890, I wrote to the Baltimore *Sun*, respecting the definition "mechanical dentist":

THE DENTISTS AND THE CENSUS.

Dr. Richard Grady writes to *The Sun* as follows:

"The prevailing idea that the gold filling and the artificial denture are as legitimate objects of barter and contract as any other tangible object of manufacture has led the census office at Washington to interpret the term

'mechanical dentists to embrace all branches of the profession except the extraction of teeth.' Mercantile relations of cost and price are capable of definite adjustment when applied to commodities of known values enhanced by labor at given rates, but professional service is amenable to no such standard. Dentistry is the science and art of medicine applied to the dental organs, and the fee is a valuation of thought and skill. The anatomy, physiology and pathology of dentistry differ in no respect from that taught in medical schools. Dental therapeutics, however, include a class of operations not taught in medical schools and not practiced in the offices of physicians and surgeons. Hence by universal consent this branch of therapeutics, under the name of dental surgery, is assigned to a special class of practitioners.

"The distinguishing feature of dental therapeutics is the art of replacement—replacement of dental *structure*, replacement of dental *organs*, which may be classified as dentistry of the chair and dentistry of the laboratory. As medicine and surgery are combined in the practice of the majority of medical men, so the two classes of prosthetic mechanism are usually practiced together, and such practice is in a large number of cases unavoidable. The element of mechanism and the necessity for the exercise of handcraft enter more or less into all special sciences. Astronomy, chemistry, pharmacy, microscopic analysis and modern medical diagnoses demand extreme accuracy of manipulation, and all great discoverers in these sciences displayed the ability not only to use, but also to invent and construct apparatus.

"The universal recognition of the great value of this element in every department of physics has given the scientific world a correct idea of the true dignity of highly educated mechanical skill—skill without which the physician's art is crippled, surgery becomes impotent and dentistry has no existence. No science or art except chemistry has been so eminently progressive in recent years as dentistry, and it is second in importance and extent of its usefulness to no specialty of the great art of healing. It requires the talent that would make a good surgeon, the talent that would make a good mechanic, and the talent that would make a good artist."

October 29, 1890, the Census office having decided adverse to the dentists, saying, "This office is strongly of the opinion . . . that such features of the profession are clearly within the limits of the census inquiry relating to productive industry, and, as such, are under the law to be included in the tables of manufactures," I wrote again to the *Sun*, discussing the new interpretation of "mechanical dentists," as follows:

THE DENTISTS AND THE CENSUS MEN.

*Messrs. A. S. Abell & Co:*

The dental profession of Baltimore is certainly under obligations to *The Sun* for the interest shown by its representatives in Washington and at home in discussing "the census and the dentists." The interpretation given by Mr. Williams to the term mechanical dentistry, namely, "That branch of the profession which includes the manufacture of artificial dentures, such as plates, crowns, caps," modifies that given September 23, 1890, by Chief of Division Boudinot to Chief Special Agent Creager: "You are informed that the term is considered to embrace all branches of the profession except the extraction of teeth." The census office is in error in claiming that "at the census of 1880 * * * opposition was uniformly withdrawn when proper explanations * * * were made to the persons averse to giving the information required."

The facts are that the superintendent of the census of 1880, upon the representation made to him by the chief special agent of New York "of the general reluctance of the profession to give the information contemplated by the schedule," and upon the presentation to him of a "protest against the novel and unjustifiable demand made for reports from dental practitioners," determined to omit from the tabulations "dentistry in *all* its branches." "If, as the result of this decision, all the schedules in the New York office were "indorsed to that effect and respectfully returned to their respective makers," where did the tenth census get the various statistical items relating to mechanical dentistry? Again, referring to the figures of that census, the number of establishments noted for Baltimore is 14, which would imply that but one in ten of 141 dentists whose names are given in the Baltimore City Directory for 1880 manufactured artificial dentures, crowns, caps, &c., whereas it is well known that such work is done by the majority of the profession.

RICHARD GRADY, M. D., D. D. S.

It having been published from Washington that, "where information was refused in the manner proposed by the Maryland dentists," the penalties provided by the census law could be resorted to; and it having been hinted that I was "a marked man" for one of the "first victims" because (as alleged) of my having originated the opposition; I again sought Dr. Twilley to ascertain if there was not some covert bias or prejudice on the part of the "powers that be;" to learn if there was any substantial reason why the action of the meeting of the Maryland State Dental Association, November 21, 1890, (to which I was invited and at which I was present) should not apply to me. (The organization had voted that "the sum of $200 be appropriated to defray the expenses of him of whom a test case is made)." The reply caused no surprise and proved the accuracy of my suspicion that there would be no aid in preventing the threatened mischief from those who, I had a right to expect, should be the first to come to my assistance; who should have said: "Yes, we'll stand by him; even if we can't approve all his acts, we won't desert him in his hour of trial; tell him we believe he is more faithfully discharging his duty to the dental profession by resisting the encroachments of the Census Bureau than he would be by submitting to its unlawful authority, for he becomes not only the vindicator and defender of his own rights but of the rights of every dentist in the community and of the well-being of the dental profession itself, because in the professions each individual assumes a trust relation to his associates and his private ends become of secondary importance." I am speaking practically not egotistically.

May I, without offence, ask you to consider that the special "victim" selected by the Census Office from among those present at the meeting referred to had nothing to do with the point at issue, because it was a *thing*—the classification of dentists as manufacturers—that was being fought? I bury all remembrance of the incident and only mention it because justice to truth demands the statement. These are Dr. Twilley's despairing words (original letter):

BALTIMORE, Nov. 22nd, 1890.

*Dear Doctor :*

I have just returned from my tramp and must confess do not feel near so good as when I started out, for then I had hopes. Now I have none. There will be no support given by one faction of the profession; of this, I am quite well assured.

Hoping all will end well, I remain truly yours,

W. S. TWILLEY.

December 30, 1890, it having been "decided that the census office would exercise the full power of the census law in order to accomplish its object" * * * "that the census officials' demand for an answer to all inquiries must be complied with" * * * that those who refuse to answer the census inquiry will be strictly prosecuted;" and Mr. Williams, a special agent of the Census Office having said to a *Sun* representative that the same questions that the Baltimore dentists object to are being put to the dentists in other cities and "so far we have not received a single complaint from any of them; we are receiving their replies every day," I, not willing to let such an official statement go unchallenged. if not uncontradicted, with painful iteration, wrote to *The Sun* as follows:

THE CENSUS AND THE DENTISTS.

Dr. Richard Grady writes to *The Sun* respecting the decision of the census office that dentists who refuse to answer the census inquiry will be prosecuted. He says: "The iteration and reiteration in Washington that 'the dentists of Baltimore are now, according to Mr. Williams's statement, the only ones who resist the purpose of the census office inquiry, prompted me to send a clipping to the editor of the *Dental Cosmos*, Philadelphia, for verification, as the Philadelphia County Dental Association had unanimously adopted resolutions instructing 'its members to decline to attempt the impossibility of making any returns.' His reply is: "As stated in the December *Cosmos*, the Philadelphia Dental Society's resolutions set forth the protest of an incorporated body, which has, I am told, formally presented its protest to the authorities at Washington and has no intention of receding from the position taken."

I grieve to say that just when the atmosphere of hope and courage created by the news to the Baltimore dentists that other cities were declining to make returns, afforded unparalleled opportunity for success, the public press, (January 14, 1891) announced from Washington that "Baltimore dentists will answer" and that Mr. Williams, special agent of the Census Office, "believes this ends the entire difficulty and that the dentists of Baltimore will no longer hesitate about furnishing the information which the census office demands." What a spectacle Baltimore's strong men present! At the first shock they fall: they refuse, then hesitate, then "saying they would never consent, consented." We were disappointed, but we were neither dismayed nor defeated yet. Nothing could have been more sudden, more startling, more unlooked for, than the "understanding reached whereby it was conceded on the part of the committee ("a committee representing the dentists of Baltimore" is the language of Supt. of Census Porter whom I am quoting, and which is misleading to put the thing politely) that it was for the good of all concerned that dentists should furnish the information which the Census Office desired." A feeling of depression, if not of indifference, almost insuperable, followed the division of sentiment among the Baltimore dentists and a diversion of their influence. The agreement which recited that "the dental profession would best serve its interests by complying with the request of the Census Office in furnishing the information called for" was fiercely denounced as an abandonment of important rights. If not intentional, it was construed as certainly, in its results, a most disastrous evasion of the views of the incorporated dental organizations of the city and State which had "resolved that all members refuse to sign the report offered by the census enumerator."

January 19, 1891, I published in *The Sun* this answer:

THE DENTISTS AND THE CENSUS.

In reference to the paper signed last week in Washington, Dr. Richard Grady said: "The three dentists who signed the paper at the census office did not voice the sentiments of the incorporated bodies of the city and State, the Association of Dental Surgeons of Baltimore City and the Maryland State Dental Association, which have unanimously adopted resolutions declining to make returns, which resolutions have never been rescinded, and that the president of the Philadelphia Dental Society has written under date of January 16 that the 'society has no intention of receding from the position taken. In no case that I am aware of has any member of the society filled out a blank of the manufacturers' census since the passage of the resolution."

I am pleased to say that in three weeks the error was atoned for, Lawyer Venable's opinion—"There is no penalty whatever for refusing to answer the questions * * * the dentists may either ignore the whole matter or answer generally that they do not operate manufacturing establishments"—having turned the scales; and February 3, 1891, the Baltimore Dental Club "resolved that the members of the Club refuse to answer the questions concerning manufactures which were presented by the Census Bureau," and the committee which made an agreement with the Census Office signed a letter which read: "We gave our signature to that paper under the impression that the Census Bureau had full power and authority to enforce dentists to fill up the usual manufacturers' blank as presented to them" * * * "We have now changed our opinion in reference to the matter, and demand a release from all obligations contained in said agreement."

I have no further personal record till the Willcox Bill was introduced in March 1892, by which a great ferment was raised, when I wrote the following which was unanimously adopted by the Maryland State Dental Association, May, 1892.

WHEREAS, a bill has been introduced in Congress, which provides that any person who shall neglect, or refuse to give information to census officers, shall be subject to a fine, "not exceeding ten thousand dollars, to which may be added imprisonment, for a period not exceeding one year," and whereas the public press reports that the bill referred to "will be used in the nature of thumbscrews and spiked boots to enforce replies," and further, that "the dentists of Baltimore, who by their refusal to answer certain questions * * * * will be the first victims of the inquisitorial machine if the bill is passed;" therefore,

*Resolved*, That this Association re-affirms the resolutions unanimously passed November, 1890, protesting against the action of the Census Bureau, in classifying dentists as manufacturers and refusing to fill out blanks.

*Resolved* further, That the chairmen of the several standing committees, as now constituted, be appointed to convey the sentiments of this Association to the Senators and Representatives of Maryland in Congress, and urge them to have the bill referred to amended so as to omit dentistry in all its branches, from the tabulations of the census.

The following editorial in the AMERICAN JOURNAL OF DENTAL SCIENCE, June, 1892, concluded my writing on the subject:

### DENTISTS AND THE CENSUS.

The April number noted that a bill had been introduced into Congress in March providing penalties for refusal to answer questions included in the schedules of the Census Office, and the May number referred to the unanimous action of the Maryland State Dental Association in appointing at its ninth annual session in May a committee to convey to the Senators and Representatives of Maryland in Congress the protest of the dentists "against the action of the Census Bureau in classifying dentists as manufacturers."

This committee acted promptly in the matter, visiting Washington on several occasions and agreed upon an amendment to the Bill to be introduced before it was reported to the Senate (as it had already passed the House of Representatives), providing that nothing in the Act "shall be construed as authority to collect statistics from professional men such as lawyers, physicians and dentists."

The House Bill, it should be stated, gave the Secretary of the Interior and his subordinates in the Census Bureau the authority to include dentists in the schedule of manufacturers, and to compel them under severe and unprecedented penalties to give detailed statements of their entire business.

The Committee was finally given a hearing before the Senate Committee on the Census, with a view to having the amendment referred to added to the bill, so that if it became a law it would not affect dentists. The matter was so effectually put not only by the representatives from Maryland, but also by committees from the District of Columbia, Pennsylvania and New Jersey, that the Superintendent of the Census agreed in writing "with the representatives of the Dental Association present at a meeting of the Committee on the Census to carry out the spirit and purposes" of the amendment.

The task is done. I did not choose the subject. I have not even desired to write upon it. If I have done a work of supererogation it is because you have invited me to the effort. I have intended to avoid misunderstanding by confining myself to written and printed matter. I have been exceedingly careful and guarded in my statements, and I believe I have said nothing against which any real exception can be taken by those acquainted with the subject. The language sounds, perhaps, laudatory and condemnatory; but not for a moment do I presume to pluck a leaf from the garland which your committee will bestow upon him who is indisputably the author of the movement, for it is richly deserved.

RICHARD GRADY.

BALTIMORE, February 6, 1895.

